# Lab meets real life: A laboratory assessment of spontaneous thought and its ecological validity

**DOI:** 10.1371/journal.pone.0184488

**Published:** 2017-09-14

**Authors:** Christine Kuehner, Annett Welz, Iris Reinhard, Georg W. Alpers

**Affiliations:** 1 Research Group Longitudinal and Intervention Research, Department of Psychiatry and Psychotherapy, Central Institute of Mental Health, Medical Faculty Mannheim, Heidelberg University, Mannheim, Germany; 2 Department of Biostatistics, Central Institute of Mental Health, Medical Faculty Mannheim, Heidelberg University, Mannheim, Germany; 3 Department of Psychology, School of Social Sciences, University of Mannheim, and Otto-Selz-Institute, University of Mannheim, Mannheim, Germany; Virginia Commonwealth University, UNITED STATES

## Abstract

People’s minds frequently wander towards self-generated thoughts, which are unrelated to external stimuli or demands. These phenomena, referred to as “spontaneous thought” (ST) and “mind wandering” (MW), have previously been linked with both costs and benefits. Current assessments of ST and MW have predominantly been conducted in the laboratory, whereas studies on the ecological validity of such lab-related constructs and their interrelations are rare. The current study examined the stability of ST dimensions assessed in the lab and their predictive value with respect to MW, repetitive negative thought (uncontrollable rumination, RUM), and affect in daily life. Forty-three university students were assessed with the Amsterdam Resting State Questionnaire (2^nd^ version) to assess ten ST dimensions during the resting state in two laboratory sessions, which were separated by five days of electronic ambulatory assessment (AA). During AA, individuals indicated the intensity of MW and RUM, as well as of positive and negative affect in daily life ten times a day. ST dimensions measured in the lab were moderately stable across one week. Five out of ten ST lab dimensions were predicted by mental health-related symptoms or by dispositional cognitive traits. Hierarchical linear models revealed that a number of ST lab dimensions predicted cognitive and affective states in daily life. Mediation analyses showed that RUM, but not MW per se, accounted for the relationship between specific ST lab dimensions and mood in daily life. By using a simple resting state task, we could demonstrate that a number of lab dimensions of spontaneous thought are moderately stable, are predicted by mental health symptoms and cognitive traits, and show plausible associations with categories of self-generated thought and mood in daily life.

## Introduction

People spend a considerable amount of time in their daily lives on thoughts unrelated to external stimuli or demands. These phenomena are referred to as”self-generated thought” (SGT), “spontaneous thought” (ST) or “mind wandering” (MW) [1–3]. Although these terms are frequently used alternately, we will use the term SGT, i.e. “the capacity for a person to generate mental contents that are not derived directly from immediate perceptual input” ([1] p. 489), as an umbrella term for both ST and MW, the term ST when describing task-free self-generated thoughts during rest, and the term MW when describing task-unrelated self-generated thoughts during external demands or activities in the following [2]. All forms of SGT are expressions of human conscious awareness. Whereas SGT can occur whether or not an individual is aware of its occurrence (i.e., with and without “meta-awareness” [4, 5], people can usually indicate if a thought was occurring in their mind, e.g., when interrupted and asked to do so at a given time point [6].

MW is defined as “a shift of attention away from a primary task toward internal information, such as memories” [7]. It is typically studied in laboratory studies using low demanding sustained attention tasks such as the Sustained Attention to Response Task (SART, [8]), a simple go/no-go task, combined with intermittent thought probes to receive a subjective measure of MW (i.e., task unrelated thoughts, TUTs [9–11]). Others collect self-report data after completion of the experimental session to avoid artificial disruptions of the time-course data [1]. MW episodes can also be captured outside the lab during daily life, by, for example, simply asking people whether they are thinking about something other than what they are currently doing [12]. Across a variety of laboratory tasks as well as in daily life, research suggests that 15% to 50% of participants’ time is spent mind wandering [1, 7, 9, 12]. Individuals often lack meta-awareness that their mind has wandered [1, 7, 13, 14], and a substantial number of MW episodes occur without intent [15, 16].

The resting state is presumed to be a period when high frequencies of ST can occur [7]. It is defined as a state of relaxed wakefulness, where the mind is free to wander as it is not actively engaged in any task [13]. Resting-state neuroimaging has revealed that ST is associated with a specific neural network, the Default Mode Network (DMN). This group of brain regions shows high activation levels during rest periods, and is especially active during periods of SGT [1, 14]. McVay and Kane [14] propose that the basic function of the DMN is the continuous evaluation of life goals and discrepancies, and that it automatically generates a stream of thought that can enter awareness as SGT episodes. Clinical research has shown that a number of mental disorders is characterized by abnormalities of DMN activity. For example, acutely depressed individuals show hyperconnectivity within DMN regions, which is linked to the tendency to ruminate [17, 18].

Studies using experimental tasks to assess associations between SGT in the lab and in daily life are rare and by now almost exclusively restricted to the investigation of MW episodes [9, 11, 19]. McVay et al. [9] combined the SART procedure with intervening thought probes and showed that those individuals who reported more MW during the SART task also endorsed more MW during daily life, particularly with worries as thought content. With a similar low-demanding lab task, Ottaviani and Couyoumdjian [19] found a positive correlation between MW in the lab and frequency of MW episodes during daily life one year later. The closeness of associations between lab-based and daily life-based measures of MW may depend on specific aspects related to these constructs, such as the complexity of the task employed in the lab, contextual features of MW measured in daily life, and intentionality of MW [15, 20]. By using the SART and the STROOP task, Marcusson-Clavertz et al. [11] found that low performance on the STROOP task in the lab predicted higher MW in daily life, but only in individuals with a low positive daydreaming style and when the difficulty of daily life activities was high. Furthermore, Kane et al. [21] showed that individuals with high working memory capacity (WMC) measured in the lab maintained on-task thoughts better and mind-wandered less in daily life, but only when the daily life context required concentration and effort. In a subsequent study [20], WMC predicted TUTs in the lab as well as MW in daily life as a function of subjects’ momentary attempt to concentrate, thereby confirming prior results. However, TUTs only marginally predicted daily life MW, whereas they significantly predicted other dimensions of SGT than MW, such as experiencing their thoughts as hardly to control or racing. The authors therefore concluded that the relation between lab-based and daily-life based MW is not robust and that research on SGT might profit from including other qualities of subjective cognitive experience beyond MW. Finally, Seli et al. [15, 16] provide evidence for the importance to distinguish between intended and unintended MW, which may differ with regard to their content, their impact on the level of motivation during a task, and other outcomes. Such a more detailed elaboration on SGT in the context of MW research is largely missing in the area of task-free ST. Concomitantly, there is a lack of studies investigating associations between ST in the lab and SGT in daily life. In this context, we could show previously that provoked DMN hyperconnectivity by negative mood induction via autobiographical material during scanning was linked to enhanced daily life rumination in remitted depressed patients [22].

While the resting-state paradigm is frequently employed in neuroimaging studies to assess task-free ST, Diaz and colleagues [23, 24] developed a retrospective measure of ST which includes a simple 5-min resting interval in the lab (inside or outside the scanner), followed by the self-administered Amsterdam Resting-State Questionnaire (ARSQ, [23]). The ARSQ quantifies ST along seven cognitive dimensions or phenotypes: “Discontinuity of Mind”, “Theory of Mind”, “Self”, “Planning”, “Sleepiness”, “Comfort”, and “Somatic Awareness”. An update of this questionnaire, the ARSQ 2.0 [24], includes the additional dimensions “Health Concern”, “Visual Thought”, and “Verbal Thought”. Diaz et al. [24] report satisfying test-retest correlations over short and long time intervals (3–32 months) and significant correlations with the personality trait of self-directedness for most ARSQ 2.0 dimensions. Stoffers et al. [25] identified associations of “Sleepiness”, “Visual Thought”, and “Discontinuity of Mind” with functional brain connectivity within visual, sensomotoric, and default mode networks during resting-state imaging. Marchetti et al. [26] demonstrated that individuals with high values on the dimension “Theory of Mind” showed higher functional connectivity in the frontal lobe and in the insula, with a dominance of the left hemisphere, suggesting that connectivity within the frontal lobes facilitates mentalizing ability. Others [27, 28] identified associations of several dimensions of the ARSQ with alpha and theta oscillations during rest and with sleep latency. These results suggest that the ARSQ assesses dimensions of ST as rather stable, trait-like characteristics and provide first indications for construct validity with respect to neural correlates of ST as assessed by this questionnaire in nonclinical populations.

The ST lab phenotypes assessed by the ARSQ 2.0 can be divided into those measuring particularities of the ST process (“Discontinuity of Mind”, “Visual” and “Verbal Thought”) and those measuring specific contents of ST. They can also be expected to vary in their degree of association with mental health indicators and cognitive traits. For example, “Discontinuity of Mind” describes a state characterized by the perceived lack of control over ones’ thoughts, thereby reflecting a dysfunctional thought process that is common to many psychological disorders. Diaz et al. [23, 24] have shown that this ST dimension was positively correlated with mental health problems and negatively with self-transcendence, a self-oriented process associated with spiritual traits or meditation. Regarding “Visual” and “Verbal” Thought, previous research has shown that worry is predominantly experienced in a more abstract-verbal form rather than in a concrete-visual imaginary form [29]. Abstract-verbal worry leads to higher anxiety than concrete-visual worry, and this reduced concreteness in thinking has been shown to exert negative effects on problem solving and emotion regulation [29]. Within the content dimensions, “Comfort” directly describes a positive state of well-being, which had a negative relationship with “Discontinuity of Mind” and with mental health problems, and a positive relationship with good sleep quality in the study by Diaz et al. [23]). “Theory of Mind”, which signalizes mentalizing properties in terms of thinking about others, is considered a functional cognitive ability that is important for social aspects in life [26]. In contrast, “Health Concern” includes worries about health and feeling ill and may therefore show a link to anxious states and traits [24]. “Sleepiness” may characterize the perception of a transient state of tiredness but also a more enduring condition of bodily or mental exhaustion which is also common in many psychological disorders. In the study by Diaz et al. [23], “Sleepiness” was linked to low mental well-being and to EEG biomarkers of low vigilance and drowsiness. Other dimensions, such as “Somatic Awareness”, appear to be more indirectly linked to mental health by constructs such as mindfulness (here: being aware of one’s bodily reactions, or somatosensory attention, cf. [30]) whereas “Planning” describes both elements of mindlessness, worries, and future orientation. Finally, similarly to “Theory of Mind”, “Self” appears to be most closely related to the concept of self-reflection, which has generally not been associated with adverse mental health outcomes [29, 31]. In sum, different associations with markers of mental health and functionality of cognitive traits can be expected for the various ST dimensions measured with the ARSQ 2.0.

Associations between SGT and *mood* have so far only been investigated within the same assessment context (e.g., during a lab session *or* during daily life) but not across contexts. In the prominent large-scale daily life experience sampling study by Killingsworth & Gilbert [12], participants reported lower levels of happiness when their minds were wandering than when they were not. Others, however [10, 19, 32, 33], found that the negative effect of MW on mood disappeared when differentiating between MW and perseverative cognition, such as rumination or worry, and posit that MW only becomes maladaptive when it takes a rigid form of perseverative cognition [10].

Particularly rumination, the tendency to repetitively and passively focus on the meanings, causes and consequences of one’s current feelings of distress, problems, and other negative aspects of the self, has been identified as a reliable risk factor for the development and course of depression [34–38], but also as a transdiagnostic risk factor for predicting various types of mainly internalising, but also externalising psychopathologies and their comorbidities [37, 39]. Momentary rumination in daily life has been shown to deteriorate mood [40] and to enhance cortisol secretion over the day [36]. Furthermore, instability of daily life rumination impacted the long-term clinical depression course in remitted depressed patients [38]. Research also suggests that not all aspects of rumination are equally problematic. For example, Treynor et al. [41] defined the “depressive brooding” and “reflective pondering” subtypes of rumination, of which only the former is considered to have detrimental effects on mood. Furthermore, particularly perceived uncontrollability of rumination (i.e., thoughts about how difficult it is to control or interrupt ruminative thoughts)—as a kind of “second order-” or “meta-”rumination—appears to be most strongly associated with depressive symptomatology [42].

Aim of the present study was to investigate the stability of subdomains of the ARSQ 2.0 as a measure of task-free rest-related ST in the lab, as well as associations of these subdomains with a restricted number of mental health indicators and cognitive vulnerability and protective traits. Due to previous research [10, 22–25, 30, 33, 35, 39] we expected a number of subdimensions (Discontinuity of Mind, Planning, Comfort, Health Concern, and Verbal Thought) to be linked to mental health indicators and to the maladaptive trait component of brooding rumination, whereas others were expected to be predicted by the more adaptive component of reflective rumination (Theory of Mind, Self) or trait mindfulness (Somatic Awareness). Furthermore, we aimed to assess the potential of ARSQ 2.0 subdomains to predict MW, perceived uncontrollability of rumination (RUM) as a form of perseverative negative cognition, and mood in daily life. We hypothesized that the more dysfunctional subdomains described above should particularly be linked to daily life RUM. Finally, we aimed to assess possible mediating effects of daily life MW and RUM on the relationship between ARSQ 2.0 lab dimensions and daily life mood. The latter aspect is of particular importance, because mediation models provide insight into the pathways by which a specific ST lab dimension, conceptualized as a trait-like distal predictor, may affect mood outcomes in daily life.

## Materials and methods

### Participants

We recruited 45 university students to take part in a study on SGT and mood in the laboratory and in daily life. Two participants had to be excluded due to excessive missing data, resulting in a final sample of 43 participants aged 19–32 (26 women, 17 men; *M*_*age*_ = 21.74 years, *SD* = 3.14). Participants were recruited using social networks and mailing lists for students of the University of Mannheim. They were compensated for their time with four hours of course credit. For the completion of at least 90% of the electronic daily life assessments, participants received an additional payment of 20 Euros as a bonus. All procedures performed in this study were in accordance with the the 1964 Helsinki declaration and its later amendments or comparable ethical standards. The experimental protocol was approved by the University of Mannheim Ethics Committee. All individual participants provided written informed consent.

### Procedure

**Fig 1:**
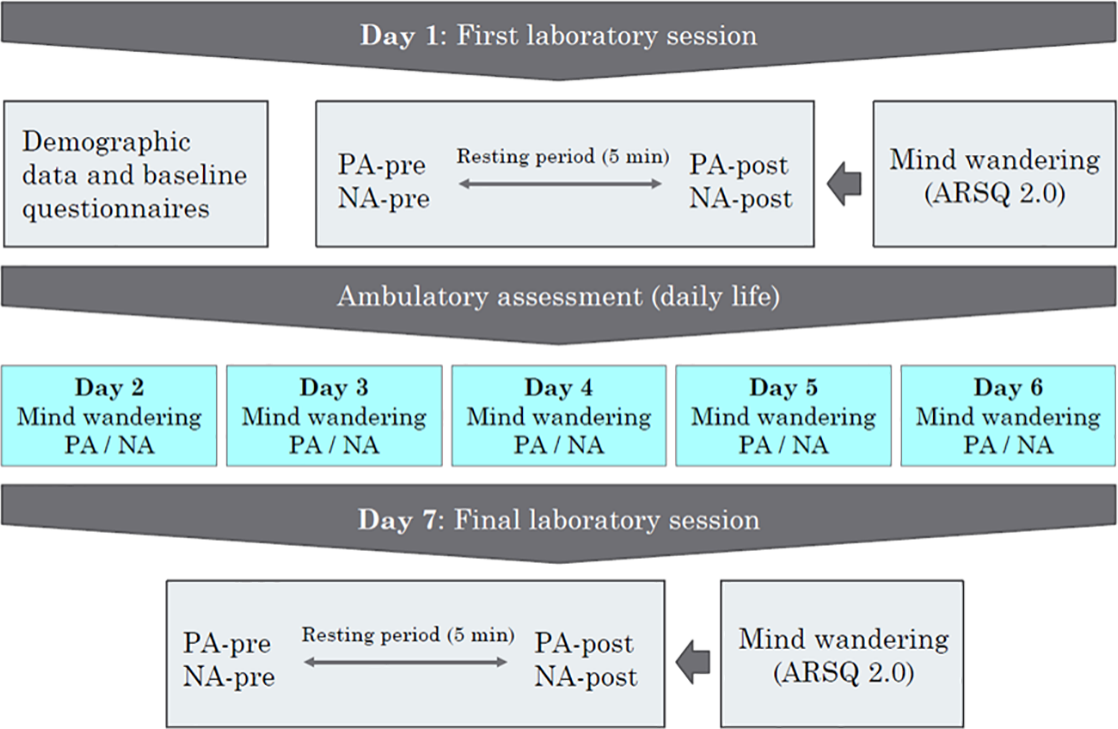
Schematic representation of the study procedure. PA = positive affect; NA = negative affect; ARSQ 2.0 = Amsterdam Resting-State Questionnaire Version 2.0.

Fig 1 shows the design of the study. The study consisted of three phases: A first laboratory session (day 1), followed by five days of ambulatory assessment (days 2–6), and a final laboratory session (day 7). On day 1, participants received written information about the study and signed the informed consent form. Demographic data was collected and participants filled out a number of baseline questionnaires. Subsequently, ST was assessed in the laboratory using the ARSQ 2.0 procedure described below.

At the end of the session on day 1, participants received detailed instructions on the use of the electronic device (smartphone) for the daily life assessment of MW, rumination, and mood taking place over the next five days (description see below). After performing the ambulatory assessment (AA), participants returned to the laboratory for a repeated measurement of ST on day 7. They also returned the smartphone, filled out a short follow-up questionnaire relating to the AA, and received their compensation.

### Questionnaires

Prior to the laboratory assessment of ST, participants completed baseline questionnaires to assess levels of depressive and anxiety symptoms as well as dispositional rumination and mindfulness.

#### Center for epidemiological studies-depression scale (CES-D)

The CES-D ([43], German version [44]) is a 20-item measure that asks to rate how often over the past week one experienced symptoms associated with depression, such as sadness, restless sleep and poor appetite. Response options range from 0 to 3 for each item (0 = rarely or none of the time, 1 = some or a little of the time, 2 = a moderate amount of time, 3 = most or all of the time). Scores range from 0 to 60, with high scores indicating greater depressive symptoms. A cut-off of 16+ has been proposed to denote individuals at heightened risk for depression [43, 45]. The CES-D has been widely used in epidemiological and clinical studies and has proved to have good psychometric properties. In the present study, the internal consistency of the CES-D was α = .85.

#### State-trait anxiety inventory, trait subscale (STAI-T)

The trait subscale of the STAI by Spielberger (German version [45]) measures anxiety as an individual difference variable, i.e., as the general tendency to react with anxiety, using 20 statements. Subjects are requested to indicate on a four point scale (1 = almost never, 2 = sometimes, 3 = often, 4 = almost always) how they generally feel. The STAI has demonstrated good reliability, concurrent and construct validity [45]. The German manual includes norm values for different subgroups, with a raw value of 56 corresponding to a T-value of 70 in young adult populations. In the present study, the internal consistency of the STAI-T was α = .89.

#### Response styles questionnaire (RSQ_10D)

Trait rumination was measured using the ten-item German version [46] of the Response Styles Questionnaire by Treynor et al. [41]. The questionnaire includes two components of rumination, namely brooding (the tendency to passively ruminate on one’s deficits and problems, e.g. “why can’t I handle things better”) and self-reflection (neutrally thinking about one’s self, e.g. “I go someplace alone to think about my feelings”). Each component is reflected by five items, with scores ranging from 1 to 20. Subjects are requested to indicate what they *generally* think or do when they feel down, sad, or depressed on a four-point scale (1 = almost never, 2 = sometimes, 3 = often, 4 = almost always). The RSQ-10 has shown good psychometric properties [46]. In the present study, the internal consistencies of brooding and reflection were *α* = .72 and *α* = .71, respectively.

#### Mindful attention and awareness scale

Trait mindfulness was measured using the German version [47] of the Mindful Attention and Awareness Scale [30]. The 15-item scale assesses the general tendency to be attentive to and aware of present-moment experiences. The wording of all items is directed towards a “mindless” disposition, for example “I rush through activities without being really attentive to them”. Subjects indicate how often they have experiences of “mindlessness” on a six-point scale (1 = almost always, 2 = very frequently, 3 = somewhat frequently, 4 = somewhat infrequently, 5 = very infrequently, 6 = almost never). Consequently, higher scores signify more mindfulness. In the present study, the internal consistency of the MAAS was *α* = .86.

### Laboratory assessments

During the laboratory sessions on days 1 and 7, participants were first instructed to assume a comfortable and relaxed sitting position in front of the computer. ST in the lab was assessed using the procedure described by Diaz et al. [24]. Experiment and instructions were presented using the open source software OpenSesame [48]. Participants were informed that the experiment is about thoughts and feelings that may be experienced during rest. They were asked to relax and to keep their eyes closed for five minutes, to move as little as possible and to try not to fall asleep. When the five minutes were up, the participants heard a beep and were allowed to open their eyes again. They were then asked to fill out the ARSQ 2.0 [24] (see below).

#### Amsterdam resting-state questionnaire

The Amsterdam Resting-State Questionnaire (ARSQ 2.0; [24]) was developed as a standardized instrument to capture ST experiences during the resting state. It allows the quantitative assessment of thoughts and feelings along ten dimensions of ST, namely “Discontinuity of Mind”, “Theory of Mind”, “Self”, “Planning”, “Sleepiness”, “Comfort”, “Somatic Awareness”, “Health Concern”, “Visual Thought”, and “Verbal Thought”. The ten-factor model of MW exhibits acceptable fit [24]. Each factor is specified by three items, resulting in 30 items in the model. The ARSQ 2.0 additionally encompasses 19 non-factor items and five items to assess response validity, resulting in a total of 54 items. The items are scored on a five-point scale (0 = completely disagree, 1 = disagree, 2 = neither agree nor disagree, 3 = agree, 4 = completely agree). Scores for each dimension are computed based on the mean of the raw responses of the respective factor items.

The item order was randomized, except for the validation items. The data was screened in accordance with the validation items, which indicated that all participants had their eyes closed during the experiment and were able to rate the statements (rating ≥ 3), and were sufficiently motivated to participate (rating ≥ 2). However, on day 1 four participants (9.3%) indicated that they had difficulty remembering their thoughts (rating ≥ 3). On day 7, this applied to two (4.7%) participants.

The German translation of the ARSQ 2.0 was made available by the authors of the original instrument (see Supporting Information S1 File).

### Ambulatory assessment

The ambulatory assessment (AA) was carried out using Motorola Moto G 2nd Generation smartphones and the software movisensXS, Version 0.6.3862 (movisens GmbH, Karlsruhe, Germany). The AA was performed over five consecutive days and always comprised a weekend in addition to three weekdays. As participants began the study on different weekdays, sequence effects were avoided since the weekend could fall in the beginning, middle or end of the assessment period.

Participants started the AA procedure on the day immediately following the first laboratory session. There were ten assessments each day, with the first assessment taking place at 9.00 a.m. and the remaining assessments taking place between 10.00 a.m. and 10.00 p.m. at random time points at least 60 minutes apart. Each assessment was announced by an acoustic signal or vibration alert, and participants were asked to immediately respond to the questions, which took two to three minutes to complete. If participants were unable to respond at the time of the signal, they could press a button to delay their response by 15 minutes. If they failed to respond to the alarm, it was repeated five times within the space of five minutes. If the participant rejected the alarm or ultimately failed to respond to it, the assessment was saved as missing. Participants were provided with written instructions regarding the electronic diary procedure.

#### Mind wandering (MW, AA)

Each time a participant was assessed by the electronic diary, they were first asked the mind-wandering question (derived from [12]): “At the time of the beep, were you thinking about something other than what you were currently doing?”. Participants responded on a seven-point scale ranging from 1 (I was completely on task) to 7 (I was completely off task).

#### Perceived uncontrollability of rumination (RUM, AA)

Second, ruminative self-focus was captured with the item “At the moment, I am stuck on negative thoughts and cannot disengage from them”. This item was rated on a scale from 1 to 7, and captured the uncontrollability facet of rumination (AA; cf. [42]).

#### Positive (PA) and negative (NA) affect (AA)

Finally, participants were asked to rate their current mood on 12 items balanced in valence (i.e. positive and negative) and arousal (i.e. the degree of activation). The items were based on the Positive and Negative Affect Schedule (PANAS; [49]) and previous experience-sampling studies [50, 51]. PA was measured using the words: cheerful, energetic, enthusiastic, satisfied, relaxed, and calm; NA was assessed with the words: upset, irritated, nervous, listless, down, and bored. Participants were asked to rate the items on a seven-point scale ranging from 1 (not at all) to 7 (very). PA and NA scores were calculated based on the sum of the respective scale items.

### Data analysis

To determine the stability of the ARSQ 2.0 ST lab dimensions, we first computed Pearson correlations of the ten dimensions assessed on days 1 and 7. We additionally used equivalence testing [52] to demonstrate mean equivalence in two dependent samples. Equivalence tests provide statistical support for the null hypothesis, that is, evidence for “no difference”. They require the definition of a minimum substantial effect, which is the lowest effect size considered as consequential. We selected Δ = .3 as a minimum substantial effect, based on Cohen’s (1988) classification of a medium effect. The SPSS custom dialog file for the dependent/paired samples equivalence test was downloaded from http://www.dr-rene-weber.de (Software & Materials), and analyses were carried out according to the instructions by Weber and Popova [52].

With regard to the other analyses, data were analyzed with Hierarchical Linear Models (HLMs), as the data exhibited a multilevel structure with assessments (level 1) nested within participants (level 2). When specifying the fixed part of the models, the respective assessment day was always entered as a fixed effect, that is either days 1 and 7 (categorical variable, coded 1 and 2) in models regarding laboratory data, or days 2 to 6 (continuous variable, coded 1 to 5) in models pertaining to daily life assessments. The daily life models also included time over the day (continuous variable, exact time since first assessment) as a fixed effect. Finally, all HLMs included a random intercept to indicate that the mean levels of the respective dependent variable may vary between individuals.

In all models that include the daily life measures of MW and RUM (i.e., predictive effects of ST lab dimensions on MW and RUM, meditation models), we used the time-lagged scores (measured at t-1) of MW and RUM and the non-lagged scores (measured at time t) of the mood outcomes. These analyses were performed with IBM SPSS Version 22.

The mediation analyses for multilevel models were conducted using the procedures described in Bauer, Preacher, and Gil [53] using a lower level mediation model (2-1-1). Thereby the effect of a level 2 predictor is mediated and the mediator is a level 1 variable. Fitting the model is done by stacking the dataset and using the procedure PROC MIXED from SAS Software Version 9.4. For calculating and testing the indirect and total effects we used a modification of the SAS macro INDTEST provided by Bauer on the Web at http://supp.apa.org/psycarticles/supplemental/met_11_2_142/met_11_2_142_supp.html. In addition, a Monte Carlo procedure implemented in this macro based on 100,000 samples yields 95% confidence intervals around the indirect and the total effect. Zero falling outside the interval indicates that the respective effect is significant.

## Results

### Descriptive analyses and compliance with ambulatory assessment

The means and standard deviations as well as the minimum and maximum values obtained on all variables assessed in the laboratory and in daily life are presented in Table 1. For descriptive purposes, Table 1 presents aggregated scores for the ten ARSQ 2.0 ST lab dimensions, averaged over the two days of laboratory assessment (day 1, day 7). Similarly, aggregated scores for MW, RUM, and PA and NA in daily life were calculated by averaging the individual scores per person over the five days of ambulatory assessment (day 2 to day 6). As shown in Table 1, the percentage of participants reporting elevated depression scores (CES-D) was 9.3%, and that of those with elevated anxiety scores was 7.0%.

**Table 1:** Descriptive statistics of the main variables assessed in the laboratory and in daily life (N = 43).

		M/N	SD/%	Min	Max
Laboratory assessments					
	Discontinuity of Mind^a^	2.11	0.75	0.17	3.50
	Theory of Mind^a^	2.30	0.72	0.50	3.67
	Self^a^	2.37	0.83	0.33	4.00
	Planning^a^	2.45	0.87	0.83	4.00
	Sleepiness^a^	1.14	0.64	0.00	2.50
	Comfort^a^	2.95	0.69	0.00	4.00
	Somatic Awareness^a^	2.45	0.90	0.50	4.00
	Health Concern^a^	0.52	0.63	0.00	2.50
	Visual Thought^a^	2.61	0.78	0.67	3.83
	Verbal Thought^a^	1.39	0.85	0.00	3.50
Ambulatory assessments					
	Mind wandering^b^	3.16	1.90	1	7
	Uncontrollable rumination^b^	1.90	1.37	1	7
	Positive affect^b^	*28*.*19*	*6*.*19*	*6*	*42*
	Negative affect^b^	*14*.*42*	*5*.*56*	*6*	*42*
Questionnaires					
	CES-D CES-D 16+^c^	9.60 4	6.14 9.3%	1 -	30 -
	STAI-T STAI-T 56+^d^	37.23 3	8.27 7.0%	23 -	61 -

Note. CES-D = Center for Epidemiological Studies–Depression Scale (German version); STAI-T = Trait scale of the State-Trait Anxiety Inventory (German version)

^a^ The ten dimensions of mind wandering were aggregated over the two assessment days.

^b^ The daily-life variables were aggregated over the five assessment days.

^c^ Raw value of 16+ indicates clinically relevant depressive symptom level [43].

^d^ Raw value of 56+ corresponds to a T-value of 70+ in the age range of young adults [45].

In total, 1.964 out of 2.150 daily life assessments (5 days * 10 assessments per day * 43 participants) were completed, resulting in a compliance rate of 91.3%. In a follow-up questionnaire after ambulatory assessment, 22 participants (51.2%) reported that the assessments were *not at all unpleasant*, 18 participants (41.9%) found them *slightly unpleasant*, and two participants (4.7%) *quite unpleasant* (one participant neglected to answer the question). When participants were asked whether they had altered their behavior due to assessments, the majority (31 participants, 72.1%) indicated that they had *almost never* done so, with only one participant (2.3%) reporting that this had *almost always* been the case.

### Stability of ST lab dimensions

Table 2 shows the retest coefficients and the results of equivalence testing between the first (day 1) and second day (day 7) assessments of the ten ARSQ 2.0 ST lab dimensions. Moderate to high retest correlations were obtained for all ST dimensions apart from Sleepiness. These results provide a first indication of the correlative stability over time for nine out of ten of the ST dimensions. Mean equivalence was shown for five of the ten dimensions: “Discontinuity of Mind”, “Self”, “Sleepiness”, “Health Concern”, and “Verbal Thought” (see Table 2).

**Table 2:** Stability of the ARSQ 2.0 lab dimensions.

	Retest-correlations^*a*^	Equivalence testing^*b*^	
ARSQ 2.0 dimension	*r*	*t*	*p*
Discontinuity of Mind	.485**	0.23	.019
Theory of Mind	.488**	0.77	.065
Self	.618**	0.37	.027
Planning	.571**	-1.17	.131
Sleepiness	.169	-0.28	.022
Comfort	.544**	1.86	.331
Somatic Awareness	.595**	3.48	.862
Health Concern	.586**	0.08	.013
Visual Thought	.371*	0.81	.069
Verbal Thought	.618**	-0.55	.040

*Note*. ARSQ 2.0 = Amsterdam Resting State Questionnaire, Version 2.0 (Diaz et al., 2014, German version).

^a^Pearson correlation coefficients between the first (day 1) and second (day 7) assessments of the ten dimensions of mind wandering of the ARSQ 2.0.

* p < .05

** p < .01

^b^Equivalence testing for mean equivalence between the first (day 1) and second (day 7) assessments of the ten dimensions of mind wandering of the ARSQ 2.0. Δ = .30 and df = 42 for each equivalence test.

### Prediction of ST lab dimensions by symptoms and trait measures

HLMs were used to assess the predictive value of depressive symptoms, trait anxiety, dispositional brooding and reflective rumination, and trait mindfulness at baseline for the ten ST lab dimensions measured at day 1 and day 7 (dependent variables). Results of these models are shown in Table 3. The table presents the simple associations of respective predictors with ST lab dimensions (i.e., separate models for each predictor, together with the fixed effect of assessment day).

**Table 3:** Hierarchical Linear Models on the Effect of Symptoms and Dispositional Traits on Mind Wandering Dimensions in the Laboratory (ARSQ 2.0, Day 1, Day7, backward regression).

	**Discontinuity of Mind**			**Theory of Mind**			**Self**			**Planning**			**Sleepiness**		
*Fixed effect*	*B*	*SE*	*p*	*B*	*SE*	*p*	*B*	*SE*	*p*	*B*	*SE*	*p*	*B*	*SE*	*p*
CES-D depression	0.04	0.02	.021	0.01	0.02	.726	0.03	0.02	.157	0.04	0.02	.044	0.02	0.02	.368
STAI anxiety	**0.04**	**0.01**	**.002**	0.01	0.01	.592	0.02	0.02	.230	**0.05**	**0.01**	**< .001**	**0.02**	**0.01**	**.048**
RSQ-10 Brooding	0.06	0.04	.121	0.03	0.04	.509	0.07	0.04	.110	0.09	0.05	.064	0.03	0.04	.486
RSQ-10 Reflection	0.02	0.04	.508	**0.09**	**0.03**	**.009**	**0.13**	**0.03**	**< .001**	0.05	0.04	.256	-0.02	0.03	.476
MAAS Mindfulness	-0.02	0.01	.025	-0.00	0.01	.686	-0.01	0.01	.653	-0.02	0.01	.056	-0.00	0.01	.738
	**Comfort**			**Somatic Awareness**			**Health Concern**			**Visual Thought**			**Verbal Thought**		
*Fixed effect*	*B*	*SE*	*p*	*B*	*SE*	*p*	*B*	*SE*	*P*	*B*	*SE*	*p*	*B*	*SE*	*p*
CES-D depression	**0.08**	**0.01**	**< .001**	**-0.04**	**0.02**	**.048**	0.02	0.02	.164	-0.02	0.02	.366	0.01	0.02	.685
STAI anxiety	-0.05	0.01	< .001	-0.02	0.02	.336	0.02	0.01	.072	-0.01	0.02	.553	0.03	0.02	.068
RSQ-10 Brooding	-0.07	0.04	.058	-0.01	0.05	.774	0.02	0.04	.614	-0.01	0.04	.785	-0.01	0.05	.804
RSQ-10 Reflection	-0.03	0.03	.311	-0.00	0.04	.986	0.00	0.03	.884	-0.02	0.04	.555	0.05	0.04	.254
MAAS Mindfulness	0.02	0.01	.052	0.02	0.01	.211	-0.00	0.01	.716	0.01	0.01	.678	-0.01	0.01	.554

*Note*. All models include random intercepts as well the assessment day as fixed effects. CES-D = Center for Epidemiological Studies–Depression Scale; STAI-T = Trait scale of the State-Trait Anxiety Inventory; RSQ-10 Brooding = Brooding subscale of the 10-item Response Styles Questionnaire; RSQ-10 Reflection = Reflection subscale of the 10-item Response Styles Questionnaire; MAAS = Mindful Attention and Awareness Scale. Models were based on 1,964 observations.

To account for possible collinearity among selected candidate predictors, we then used backward elimination of predictors, by stepwise removing the predictor with the least significant contribution to the model and retained all predictors in the model that significantly (*p* < .05) contributed to the respective ARSQ 2.0 domain. Each stepwise regression analysis resulted in only one remaining significant (p < .05) predictor per ST lab domain. Respective significant predictors are marked in bold in Table 3.

According to the multiple regression analyses using backward selection, five out of the ten ARSQ 2.0 dimensions were significantly predicted by depressive or anxiety symptom scores. Higher symptom scores of depressive or anxiety symptoms predicted higher levels of “Discontinuity of Mind”, “Planning”, and “Sleepiness”, and lower levels of “Comfort” and “Somatic Awareness” (*ps* < .05). Furthermore, lower levels of dispositional mindfulness predicted higher levels of “Discontinuity of Mind” (*p* < .05) in the simple regression analysis, but this predictor was removed during the backward procedure. “Theory of Mind” and “Self” were neither linked to symptom scales nor to brooding or mindfulness. In contrast, higher levels of these ST lab dimensions were significantly predicted by higher levels on the reflection subscale of rumination (*p*s < .01). Finally, “Health Concern”, “Verbal Thought”, and “Visual Thought” were not significantly predicted by any symptom or cognitive trait measure (all *p*s >.05).

### Effects of ST lab dimensions on MW, rumination, and mood in daily life

In a next step, HLMs were used to determine whether ST in the lab predicted MW, RUM, and mood in daily life. Here, the ten ST lab dimension assessments of the lab day preceding the AA days (day 1) were used as predictors. MW, RUM (measured at time t-1), and PA and NA (measured at time t) in daily life served as dependent variables in separate models. All models included random intercepts as well as time over the day and the assessment day as fixed effects (see Method section).

As shown in Table 4, a mixed pattern of associations of ST lab dimensions with cognitive and affective processes in daily life emerged. First, three lab dimensions, namely “Theory of Mind”, “Somatic Awareness”, and “Visual Thought”, consistently yielded negative results, i.e., did not predict any of the daily life measures (*ps* >.05). Second, high levels of “Comfort” clearly predicted positive daily life cognitive and mood outcomes (low levels of RUM and NA, and high levels of PA, *p*s ≤.001). Third, higher levels of “Self” predicted higher levels of MW and RUM (*p*s < .05), and higher levels of “Verbal Thought” predicted higher levels of RUM (*p* < .05), but none of these dimensions predicted PA or NA in daily life (*p*s >.05). Finally, the remaining dimensions, namely “Discontinuity of Mind”, “Planning”, “Sleepiness”, and “Health Concern” were prospectively linked to higher amounts of MW and/or of RUM episodes, and to low PA and/or high NA (*p*s < .05) in daily life.

**Table 4:** Effects of ARSQ 2.0 MW dimensions on mind wandering, rumination, and positive and negative affect in daily life.

	Mind wandering (lagged, AA)			uncontrollable rumination (lagged, AA)			positive affect (AA)			negative affect (AA)		
Fixed effect												
	B	SE	p	B	SE	p	B	SE	p	B	SE	p
ARSQ2.0 dimensions (lab):												
Discontinuity of Mind	0.28	0.12	.021	0.24	0.14	.103	-1.46	0.59	.016	1.17	0.64	.073
Theory of Mind	0.22	0.14	.120	-0.08	0.16	.625	1.06	0.68	.127	-1.00	0.73	.177
Self	0.26	0.11	.024	0.32	0.13	.019	-0.31	0.58	.598	0.59	0.61	.341
Planning	0.23	0.11	.043	0.22	0.13	.099	-1.29	0.53	.020	0.97	0.58	.103
Sleepiness	0.28	0.12	.028	0.42	0.14	.004	-0.82	0.63	.202	1.79	0.63	.006
Comfort	-0.27	0.15	.086	-0.56	0.16	.001	2.76	0.67	< .001	-2.85	0.72	< .001
Somatic Awareness	-0.14	0.12	.235	0.01	0.14	.957	0.84	0.58	.157	-0.58	0.62	.354
Health Concerns	0.05	0.17	.766	0.63	0.17	.001	-1.20	0.82	.145	1.94	0.83	.025
Visual Thought	-0.11	0.12	.365	-0.21	0.13	.128	0.85	0.57	.145	-0.96	0.60	.116
Verbal Thought	0.23	0.12	.061	0.30	0.14	.039	-0.90	0.61	.147	1.11	0.63	.088

*Note*. All models included random intercepts as well as time over the day and the assessment day as fixed effects. Models were based on 1.759 (originally 1,964) observations.

### Mediation analyses

For those ST lab dimensions providing significant (*p* < .05) effects on both cognitive (MW and/or RUM) and affective (PA and/or NA) outcomes in daily life (i.e., “Discontinuity of Mind” and “Planning” on MW and PA; “Sleepiness” on MW, RUM, and NA; “Comfort” on RUM, PA and NA, and “Health Concern” on RUM and NA, see Table 4), we fitted HLM mediation models with PA or NA at time t as dependent variable, the respective ARSQ 2.0 lab dimension as predictor, and the cognitive daily life variable (MW or RUM) as time t-1 mediator in separate analyses. Again, all models included random intercepts as well as time over the day and the assessment day as fixed effects. A Monte Carlo procedure based on 100,000 samples was used to calculate a 95% confidence interval around the indirect and the total effect. Zero falling outside the interval indicates that the respective effect is significant. Results are shown in Table 5 (with MW as mediator) and Table 6 (with RUM as mediator).

**Table 5:** Mediation effects of daily life mind wandering on the associations between ARSQ 2.0 ST lab dimensions and daily life mood.

	Positive affect				Negative affect			
Fixed effect	B	SE	p	Monte Carlo 95% CI	B	SE	p	Monte Carlo 95% CI
DOM (lab)→MW (AA)	0.31	0.12	.015					
MW (AA) →mood (AA)	-0.07	0.07	.320					
DOM (lab) →mood (AA)	-1.47	0.61	.020					
Indirect effect	-0.02	0.03	.385	-0.08; 0.02				
Total effect	-1.49	0.61	.014	-2.72; -0.33				
PLAN (lab)→MW (AA)	0.24	0.11	.040					
MW (AA) →mood (AA)	-0.07	0.07	.319					
PLAN (lab) →mood (AA)	-1.27	0.56	.028					
Indirect effect	-0.02	0.02	.407	-0.07; 0.07				
Total effect	-1.28	0.56	.022	-2.40; -0.22				
SLEEP (lab)→MW (AA)					0.32	0.13	.017	
MW (AA) →mood (AA)					0.04	0.06	.531	
SLEEP (lab) →mood (AA)					1.82	0.65	.008	
Indirect effect					0.01	0.02	.571	-0.03; 0.06
Total effect					1.83	0.65	.005	0.55; 3.12

*Note*. All models included random intercepts as well as time over the day and the assessment day as fixed effects. DOM = Discontinuity of Mind, PLAN = Planning, SLEEP = Sleepiness (ARSQ 2.0 dimensions), MW = mind wandering, mood = positive or negative affect, as appropriate, lab = measured in the laboratory session (day 1), AA = measured by ambulatory assessment (days 2–6).

**Table 6:** Mediating effects of uncontrollable rumination in daily life on the associations between ARSQ 2.0 ST lab dimensions and daily life mood.

	Positive affect				Negative affect			
Fixed effect	B	SE	p	Monte Carlo 95% CI	B	SE	p	Monte Carlo 95% CI
SLEEP (lab)→RUM (AA)					0.43	0.14	.003	
RUM (AA) →mood (AA)					0.84	0.09	< .0001	
SLEEP (lab) →mood (AA)					1.50	0.57	.013	
Indirect effect					0.36	0.12	.004	0.13; 0.61
Total effect					1.86	0.65	.005	0.56; 3.15
COMFT (lab)→RUM (AA)	-0.57	0.17	.002		-0.57	0.17	.002	
RUM (AA) →mood (AA)	-0.72	0.12	< .0001		0.84	0.09	< .0001	
COMFT (lab)→mood (AA)	2.36	0.65	.0008		-2.45	0.66	.0006	
Indirect effect	0.41	0.14	.003	0.16; 0.72	-0.48	0.15	.002	-0.78;-0.12
Total effect	2.77	0.71	< .0001	1.36; 4.12	-2.93	0.75	< .0001	-4.42;-1.45
HEALTH (lab)→RUM (AA)					0.67	0.17	.0004	
RUM (AA) →mood (AA)					0.83	0.09	< .0001	
HEALTH (lab) →mood (AA)					1.33	0.77	.092	
Indirect effect					0.56	0.16	.0004	0.27; 0.88
Total effect					1.89	0.88	.031	0.15; 3.62

*Note*. All models included random intercepts as well as time over the day and the assessment day as fixed effects. DOM = Discontinuity of Mind, SLEEP = Sleepiness, COMFT = Comfort, HEALTH = Health Concerns (ARSQ 2.0 dimensions), RUM = uncontrollable rumination, lab = measured in the laboratory session (day 1), AA = measured by ambulatory assessment (days 2–6)

As shown in Table 5, the indirect effect of ST lab dimensions on daily life mood mediated by MW in daily life was non-significant in all models. Furthermore, “Discontinuity of Mind” and “Planning” demonstrated a significant direct effect on PA, and “Sleepiness” on NA, and the respective significant total effects were not increased by the inclusion of the indirect effect via MW in these models. Therefore, the significant associations between “Discontinuity of Mind”, “Planning”, and “Sleepiness” with mood in daily life were not mediated by daily life MW.

A totally different picture emerged for RUM (see Table 6). Here, the effect of the mediator RUM and the corresponding indirect effect of ST lab dimensions via RUM was significant in all models (all *p*s < .01), and the total effect in the respective models was larger when the indirect effect was accounted for than the direct effect of the respective lab dimension on daily life mood. Thereby, the association of “Comfort” with PA and NA, and the association of “Sleepiness” and “Health Concern” with NA in daily life were significantly mediated by lagged daily life RUM.

## Discussion

The present study aimed to evaluate the stability and ecological validity of a laboratory resting state ST procedure. For this purpose, we employed a recently developed questionnaire, the ARSQ 2.0 [24], to assess several ST dimensions during the resting state in two laboratory sessions, which were separated by five consecutive days of electronic ambulatory assessment in daily life.

### Stability of ST lab dimensions

Overall, ST dimensions assessed in the lab appeared to be rather stable over time. We found that the vast majority of the ARSQ 2.0 dimensions exhibited moderate to high retest correlations over a one-week interval. Our results are therefore consistent with findings of stable retest correlations over short [23, 25] and longer intervals of up to 32 months [24] with the ARSQ 2.0. We additionally used equivalence testing [52] and identified equivalence for “Discontinuity of Mind”, “Self”, “Sleepiness”, “Health Concern”, and “Verbal Thought”. This suggests that the degree to which individuals are concerned with these aspects during ST is stable across time. With regard to the other ST dimensions, it is possible that we could not show equivalence because the sample size was too small [52].

### Prediction of ST lab dimensions by mental health symptoms and cognitive traits

Our analyses revealed that five out of the ten ST lab dimensions were predicted by levels of depressive and/or anxiety symptoms. Individuals scoring high on one of these symptom scales reported *higher* levels of “Discontinuity of Mind”, Planning”, and “Sleepiness”, and *lower* levels of “Comfort” and “Somatic Awareness” during the 5-min resting interval in the lab. These results corroborate those by Diaz et al. [23, 24] showing that a number of phenotypes of resting state cognitions assessed by the ARSQ 2.0 are associated with mental health-related symptoms. In the simple analysis, “Discontinuity of Mind” was furthermore predicted by dispositional mindfulness. Individuals scoring low in dispositional mindfulness reported less control over their thoughts, i.e. a higher tendency to have busy thoughts, rapidly switching thoughts, and difficulties holding on to one’s thoughts during the lab resting-state session. The construct of mindfulness has up to now mainly been contrasted with MW and the two have been proposed to represent roughly opposing constructs [54]. Trait mindfulness has been shown to negatively correlate with several indicators of MW such as trait mind wandering, self-reported task-unrelated thoughts and SART errors in a lab study [54], and Ottaviani & Couyoumdjian [19] showed trait mindfulness to be inversely related to MW in daily life one year later. In the context of ST, Diaz et al. [24] showed that specifically the “Discontinuity of Mind” dimension was negatively correlated to the construct of “self-transcendence”, a self-oriented process associated with spiritual traits such as meditation. Interestingly, both the MAAS, used as a measure of mindfulness in the present study, and the “Discontinuity of Mind” subfacet of the ARSQ 2.0 appear to most closely capture the “nondistractability” versus “distractability” facets of mindfulness and SGT, respectively, making the particular association between these constructs plausible [54]. However, in the multiple model, dispositional mindfulness showed no independent contribution to the explanation of “Discontinuity of Mind”, probably due to the former’s overlap with indicators of mental health. This is consistent with prior research showing that mindfulness–measured as a trait or in the context of mindfulness-based interventions–has a strong link to health and psychological well-being [30, 55].

The fact that we investigated a nonclinical sample may have influenced the associations between habitual symptoms/traits and ST lab phenotypes. This may especially be true for “Health Concern”, originally included to capture the extreme preoccupation with health status often identified in clinical samples [24], for which we identified no significant effect of anxiety symptom levels. As we observed particularly low levels on this lab dimension in the present study, it is possible that future research with high risk or clinical samples may instead detect a stronger association with mental health symptomatology, particularly with anxiety symptoms.

Two further ST lab phenotypes, namely “Theory of Mind” and “Self”, were not predicted by mental health symptoms but instead by the RSQ-10 reflection subscale, thereby confirming our hypothesis that these MW dimensions appear to be linked to a more functional disposition of self-reflection [41].

Finally, vivid and concrete visual mental imagery—in contrast to more abstract inner verbal thought processing—has been found to be related to positive mental health and reduced depressive symptoms [56]. However, none of these lab dimensions were substantially predicted by any of the symptom or cognitive trait measures in the present study. Apart from the rather low intensity of Visual Thought reported in this study, this ST lab dimension could represent a more heterogeneous phenotype with regard to the possible experience of positive and/or negative images and past versus future-oriented visual thoughts, with all of these aspects showing differential associations with positive and negative mental health aspects [56].

### Effects of ST lab dimensions on MW, rumination, and mood in daily life

Three out of the ten ST lab dimensions (“Theory of Mind”, “Somatic Awareness”, and “Visual Thought”) did not predict any daily life measure of MW, RUM, or mood. Therefore, these dimensions—or their operationalization by the ARSQ 2.0—are either unrelated to respective daily life experiences per se or their capacity to predict similar constructs across settings is short-termed. Furthermore, the dimensions “Self” and “Verbal Thought” predicted daily life cognitions, but not mood. The remaining resting-state ST phenotypes, namely “Discontinuity of Mind”, “Planning”, “Sleepiness”, “Comfort”, and “Health Concern”, significantly predicted daily life cognitions *and* mood.

Among these, only “Comfort”, was clearly linked to positive daily life outcomes. Individuals who indicated that they felt comfortable, relaxed and happy during the rest session in the lab reported low levels of RUM and NA, and high levels of PA in daily life. Thereby, this lab dimension of ST was strongly connected with daily life indicators of low cognitive vulnerability and of good psychological health. Together with the fact that “Comfort” scores were predicted by low symptom levels, these findings confirm results by Diaz et al. [23], but also extend previous research by demonstrating prospective ecological validity of this dimension, indicating its trait-like properties.

In contrast, the sense of having little control over one’s thoughts (i.e. higher scores on “Discontinuity of Mind”) seems to translate to a greater incidence of MW and low mood in daily life, thereby supporting and expanding previous findings by indicating proposed that MW reflects the processing of thoughts with high personal salience [57]. Indeed, Andrews-Hanna et al. [58] identified *Personal Significance* as one of three major dimensions for the classification of thought content during SGT, which encompasses, for example, thoughts characterized as self-relevant and goal-oriented, thereby corresponding well to this ST dimension. Furthermore, “Self” predicted RUM, but not PA and NA. While the latter could be expected due to the identified connection of this ST dimension with a more dysfunctional ST phenotype connected with negative daily life experiences [23, 24].

Individuals whose thoughts in the laboratory focused on their own behavior and feelings (i.e. higher scores on “Self”) also engaged in more MW and RUM in daily life. The former is consistent with prior observations with regard to the content of MW episodes. For example, it has been a more adaptive component of habitual self-reflection [29, 41], its strong positive association particularly with RUM during daily life was at first sight counter-intuitive. However, in the study by Diaz et al. [24], this ARSQ 2.0 dimension, as most others, was negatively correlated with Cloninger et al.´s [59] temperamental factor of self-directedness, the ability to govern behavior according to situational demands and to adapt and regulate one’s behavior to fit situations in accord with his/her chosen goals. Self-directedness has been described as a marker of executive functions that could protect individuals from depression [60]. In the study by Diaz et al. [24], “Self” was also negatively related to persistence, a tendency towards self-confidence and perseverance. Altogether, our results suggest that while “Self” is linked to negative perseverative thought during daily life, this does at the same time not appear to affect mood, thereby suggesting the involvement of protective mechanisms, which should be clarified in future research.

Individuals whose thoughts revolved around future plans and problem solving (i.e., those with higher scores on “Planning”) engaged in more MW in daily life. Stawarczyk et al. [61] found that thoughts during MW were temporally and functionally oriented towards the future, especially when participants were induced to think about personal goals before completing a mind-wandering task. Additionally, MW facilitates the integration of past and present experiences for the purposes of future planning [6]. Finally, Song and Wang [62] argued that MW episodes are strongly associated with one’s self and plans to cope with upcoming events, which also supports our observation that high levels of “Planning” during ST in the lab have an impact on MW in daily life. However, as mentioned above, “Planning” was also predicted by higher symptom scores of anxiety, pointing to a dysfunctional component of this MW lab dimension. Similarly, Diaz et al. (2014) identified negative associations with self-directedness and cooperation. While “Planning” failed to predict RUM in our study, it was linked to lower PA in daily life.

Individuals who reported more health-related concerns during rest experienced more frequent periods of RUM as well as higher levels of NA during daily life. In contrast, “Health Concern” was not significantly related to trait anxiety. As shown earlier, this ST lab dimension reflects psychopathological aspects characteristic for a number of clinical populations [24]. It will be important to investigate the longer-term ecological validity of this dimension in clinical samples with different psychopathology in future research.

High levels of sleepiness in the lab also predicted more MW, RUM, and NA in daily life. Previous studies have provided evidence for a positive association between MW and sleepiness [9, 10, 63] as well as poor sleep quality [63]. Furthermore, Ottaviani and Couyoumdjian [19] found that MW during the day significantly predicted difficulties falling asleep the following night. Research on sleep deprivation has demonstrated that lack of sleep impairs attention and working memory, as well as cognitive performances that depend on the prefrontal cortex, such as executive control [64]. EEG evidence also indicated that particularly MW shares similarities with states of drowsiness, characterized by decreased alertness and vigilance [65], letting the authors conclude that sleep deprivation might lead to more MW during the day. Our results provide further evidence indicating that sleepiness may promote MW, but also more specific negative cognitive (RUM) and affective outcomes in daily life. These identified longer-term negative effects of sleepiness in the lab on momentary experiences assessed days later are noteworthy; they suggest that this lab dimension describes a more enduring unfavorable condition rather than a merely short-term, transient state of tiredness.

In contrast to “Visual Thought”, the “Verbal Thought” domain predicted more RUM during daily life. Negative repetitive thoughts such as rumination and worry have been characterized by a more abstract-verbal than concrete-visual imagery form, and have been linked to impaired problem solving and affect regulation [29]. Thereby, the present results provide evidence for the ecological validity of the “Verbal Thought” dimension of the ARSQ 2.0.

### Mediation effects of daily life cognitions

Finally, multilevel mediation models showed that the associations of a number of ST lab dimensions, namely “Comfort”, “Sleepiness”, and “Health Concern”, with daily life mood were substantially accounted for by the extent to which individuals tend to brood over negative thoughts during daily life which they perceive as uncontrollable. In other models, we observed that MW *per se* did not exhibit effects on the relationship between ST lab dimensions and daily life mood. These results clearly highlight the necessity to account for thought contents when assessing the functional outcomes of MW (cf. [66]) and to differentiate between MW and negative perseverate cognition such as rumination [10, 32, 33]. In this context, it has to be considered that MW is a broad construct comprising heterogeneous phenomena that may include adaptive and maladaptive components. Recent studies therefore recommend to assess more specific subqualities of MW (and other SGTs), such as intentionality, time orientation, intrusiveness, and more specific contents [15, 16, 20]. The missing mediation effect of MW in our study may have resulted from a lack of specification of possible subqualities of MW. In contrast, the association between a number of ST lab dimensions with daily life mood was clearly mediated by perseverative negative thought (RUM), characterized by more specific negatively valenced thought contents, which may themselves be the product of ST or MW in daily life. Thereby, the results of the ambulatory assessment study part provide external validation for (mal-)adaptive aspects of specific ST categories as assessed with the ARSQ 2.0.

## Conclusion

In conclusion, our data support the assumption that laboratory assessments of ST components with the ARSQ 2.0 show acceptable stability as well as meaningful associations with individual differences in mental health symptoms and cognitive traits. Furthermore, we demonstrated ecological validity for a number of ST lab dimensions by establishing a link between specific thought process and thought content components of spontaneous thought episodes in the lab and the extent of MW, negative perseverative thought, and mood in daily life, and by identifying significant mediational effects of dysfunctional daily life cognition on the association between a number of ST lab dimensions and mood. Thereby, our results support the view that while unconstrained ST during rest (as assessed by the ARSQ 2.0) and task-unrelated thoughts during external demands or activities (MW) may be differentiated, they share important features, such as being decoupled from ongoing perceptual input and internally focused, being supported by the same underlying mechanism (unguided subject driven mental states), and being connected to identical or overlapping neural networks such as the DMN [1, 2, 67].

With regard to the observed mediation effect of RUM, the present study is the first showing a probable pathway by which more distal trait-like ST dimensions of the ARSQ 2.0 may affect mood, and particularly NA, in daily life. This finding is also in accordance with the conceptual Research Domain Criteria Project (RDoC) framework [68], in which rumination is included as a key component of the *Negative* Valence Systems construct of Loss [69].

In a next step, the investigation of ARSQ 2.0 dimensions in clinical compared to non-clinical populations will allow to shed further light on the associations of these ST lab phenotypes with mental health indicators, vulnerability traits, and daily life experiences. In this context, the ARSQ 2.0 could possibly help to target maladaptive aspects of ST during the resting state, thereby identifying susceptibility towards negative mental health conditions and cognitive vulnerability, and could be used as an outcome measure in interventions aiming at reducing spontaneous maladaptive thought (cf.[70]). A further step in assessing the construct validity of respective ST phenotypes would be to examine their associations with more basic psychological and neurobiological functioning in clinical and in high-risk groups across traditional diagnostic boundaries, e.g., within the RDoC framework [68].

## Limitations

The study had some limitations which we want to acknowledge. First, the ARSQ 2.0 [24] only allows the retrospective assessment of participants’ thoughts and feelings during ST episodes and, thus, could be subject to recall bias. With this instrument, it would have been unfeasible to interrupt participants’ five minutes of rest to concurrently assess the thought content of ST. As participants completed the ARSQ 2.0 promptly after the resting period, we believe that we obtained satisfactorily valid measurements of the thought content of ST.

Second, the sample was restricted to university students. Therefore, our results may not generalize to other populations.

Third, and particularly important, the sample size was only moderate. This may have resulted in some non-significant or marginally significant results due to a lack of statistical power in the present study. Connected herewith, we did not generally correct for multiple testing in the present study. This was on the one hand due to the fact that we had established preliminary a priori hypotheses regarding the (dys-)functionality of specific subdomains of the ARSQ and their possible associations with symptoms, cognitive traits, and subjective daily life experiences. Furthermore, we considered possible multicollinearity of candidate predictors for the ten ST lab dimensions by using backward elimination of nonsignificant predictors in our regression analyses. Nevertheless, the investigation of larger, more representative samples is clearly warranted to replicate the present findings. In this context, larger study samples would also allow to investigate possible moderators on the relationship between ARSQ 2.0 lab dimensions and cognitive and affective daily life measures. For example, research has shown that mood worsening after MW is particularly seen in individuals scoring high in neuroticism [71] or trait negative affect [2], cognitive reactivity, and brooding [72], and low in dispositional mindfulness [72].

Finally, some attention should be paid to potential reactivity effects in ambulatory assessment. The large number and frequency of assessments in daily life may have been a source of irritation and increased the burden on participants. However, there is generally little evidence for measurement reactivity in ambulatory assessment studies [73], and our participants showed a high rate of compliance. Therefore, we conclude that neither the participants’ mood nor the extent of MW in daily life was substantially biased by the five days of ambulatory assessment.

## Supporting information

S1 Text German version of the ARSQ 2.0. (DOCX)

S1 File PLOS ONE MindWandering-personlevel n = 43.sav. (SAV)

S2 File PLOoS ONE MindWandering-personperiod_2rows.sav. (SAV)

S3 File PLOoS ONE MindWandering-personperiod_50rows.sav. (SAV)

S4 File PLOS ONE CK_MW-Mediation_personperiod.sas7bdat. (SAS7BDAT)
