# Supplementary material for: Lab meets real life: A laboratory assessment of spontaneous thought and its ecological validity
Source: PLoS One. 2017 Sep 14;12(9):e0184488. doi: 10.1371/journal.pone.0184488 (PMC5598976; doi:10.1371/journal.pone.0184488)
Supplement: S1 Text — (DOCX) [file pone.0184488.s001.docx]

**S1 German Version of the ARSQ 2.0^1^**

| PBID |  |  |  |  |  |  | Datum: |  |  |  |  |  |  |  |  |  |
| --- | --- | --- | --- | --- | --- | --- | --- | --- | --- | --- | --- | --- | --- | --- | --- | --- |
|  |  |  |  |  |  |  |  | T. |  |  | M. |  |  | J. |  |  |
|  |  |  |  |  |  |  |  |  |  |  |  |  |  |  |  |  |

Im folgenden Fragebogen werden Ihnen Fragen über die Gedanken und Gefühle präsentiert, die Sie während der Ruhephase hatten.

Bitte geben Sie an, wie sehr Sie den folgenden Aussagen zustimmen:

**1. Absolut nicht zutreffend “--“**

**2. Nicht zutreffend “-“**

**3. Weder zutreffend noch nicht zutreffend “/“**

**4. Zutreffend “+”**

**5. Stark zutreffend “++”**

|  |  | **--** | **-** | **/** | **+** | **++** |
| --- | --- | --- | --- | --- | --- | --- |
| 1. | Ich dachte an Dinge, die ich noch erledigen muss. | 1 | 2 | 3 | 4 | 5 |
| 2. | Ich fühlte mich müde. | 1 | 2 | 3 | 4 | 5 |
| 3. | Ich hatte lebhafte Gedanken. | 1 | 2 | 3 | 4 | 5 |
| 4. | Ich fühlte mich wohl. | 1 | 2 | 3 | 4 | 5 |
| 5. | Ich dachte in Wörtern. | 1 | 2 | 3 | 4 | 5 |
| 6. | Ich dachte an andere Menschen. | 1 | 2 | 3 | 4 | 5 |
| 7. | Ich dachte an mein Verhalten. | 1 | 2 | 3 | 4 | 5 |
| 8. | Ich dachte an meine Atmung. | 1 | 2 | 3 | 4 | 5 |
| 9. | Ich dachte an meine Gesundheit. | 1 | 2 | 3 | 4 | 5 |
| 10. | Ich dachte in Bildern. | 1 | 2 | 3 | 4 | 5 |
| 11. | Ich führte Gespräche mit mir selbst. | 1 | 2 | 3 | 4 | 5 |
| 12. | Ich fühlte mich krank. | 1 | 2 | 3 | 4 | 5 |
| 13. | Ich war mir meines Körpers bewusst. | 1 | 2 | 3 | 4 | 5 |
| 14. | Ich fühlte mich schläfrig. | 1 | 2 | 3 | 4 | 5 |
|  |  | **--** | **-** | **/** | **+** | **++** |
| 15. | Ich dachte an das Lösen von Problemen. | 1 | 2 | 3 | 4 | 5 |
| 16. | Ich hatte schnell wechselnde Gedanken. | 1 | 2 | 3 | 4 | 5 |
| 17. | Ich fühlte mich entspannt. | 1 | 2 | 3 | 4 | 5 |
| 18. | Ich versetzte mich in die Lage anderer. | 1 | 2 | 3 | 4 | 5 |
| 19. | Ich dachte an meine Gefühle. | 1 | 2 | 3 | 4 | 5 |
| 20. | Ich stellte mir Ereignisse vor. | 1 | 2 | 3 | 4 | 5 |
| 21. | Ich dachte an meinen Herzschlag. | 1 | 2 | 3 | 4 | 5 |
| 22. | Ich dachte an die Zukunft. | 1 | 2 | 3 | 4 | 5 |
| 23. | Ich dachte an Menschen die ich mag. | 1 | 2 | 3 | 4 | 5 |
| 24. | Ich hatte Schwierigkeiten wachzubleiben. | 1 | 2 | 3 | 4 | 5 |
| 25. | Ich dachte über mich selbst nach. | 1 | 2 | 3 | 4 | 5 |
| 26. | Ich fühlte mich glücklich. | 1 | 2 | 3 | 4 | 5 |
| 27. | Ich spürte Schmerzen. | 1 | 2 | 3 | 4 | 5 |
| 28. | Ich stellte mir Umgebungen vor. | 1 | 2 | 3 | 4 | 5 |
| 29. | Ich stellte mir vor, mit mir selbst zu sprechen. | 1 | 2 | 3 | 4 | 5 |
| 30. | Ich konnte meine Gedanken nur mühsam festhalten. | 1 | 2 | 3 | 4 | 5 |
| 31. | Ich empfand die Sitzung als angenehm. | 1 | 2 | 3 | 4 | 5 |
| 32. | Ich fühlte mich unruhig. | 1 | 2 | 3 | 4 | 5 |
| 33. | Ich hatte Gedanken, die ich nicht mit anderen teilen würde. | 1 | 2 | 3 | 4 | 5 |
| 34. | Ich dachte an angenehme Dinge. | 1 | 2 | 3 | 4 | 5 |
| 35. | Ich fühlte nichts. | 1 | 2 | 3 | 4 | 5 |
| 36. | Ich dachte an meine Arbeit/mein Studium. | 1 | 2 | 3 | 4 | 5 |
|  |  | **--** | **-** | **/** | **+** | **++** |
| 37. | Meine Gedanken blieben ungefähr gleich während der Sitzung. | 1 | 2 | 3 | 4 | 5 |
| 38. | Ich fühlte mich ungefähr gleich während der Sitzung. | 1 | 2 | 3 | 4 | 5 |
| 39. | Ich langweilte mich. | 1 | 2 | 3 | 4 | 5 |
| 40. | Ich hatte negative Gefühle. | 1 | 2 | 3 | 4 | 5 |
| 41. | Ich dachte an Geräusche in meiner Umgebung. | 1 | 2 | 3 | 4 | 5 |
| 42. | Ich hatte oberflächliche Gedanken. | 1 | 2 | 3 | 4 | 5 |
| 43. | Ich dachte an Gerüche in meiner Umgebung. | 1 | 2 | 3 | 4 | 5 |
| 44. | Ich dachte an die Vergangenheit. | 1 | 2 | 3 | 4 | 5 |
| 45. | Ich dachte an das Ziel des Experiments. | 1 | 2 | 3 | 4 | 5 |
| 46. | Ich dachte an die Gegenwart. | 1 | 2 | 3 | 4 | 5 |
| 47. | Ich hatte tiefgründige Gedanken. | 1 | 2 | 3 | 4 | 5 |
| 48. | Ich dachte an nichts. | 1 | 2 | 3 | 4 | 5 |
| 49. | Ich hatte meine Gedanken unter Kontrolle. | 1 | 2 | 3 | 4 | 5 |
| 50. | Ich hatte meine Augen geschlossen. | 1 | 2 | 3 | 4 | 5 |
| 51. | Ich fühlte mich motiviert teilzunehmen. | 1 | 2 | 3 | 4 | 5 |
| 52. | Ich kann mich nur mühsam an meine Gedanken erinnern. | 1 | 2 | 3 | 4 | 5 |
| 53. | Ich kann mich nur mühsam an meine Gefühle erinnern. | 1 | 2 | 3 | 4 | 5 |
| 54. | Ich war imstande, diese Aussagen zu bewerten. | 1 | 2 | 3 | 4 | 5 |
| * |  |  |  |  |  |  |

**AUSWERTUNG**

| **ARSQ 2.0 Dimension** | **Items** |
| --- | --- |
|  |  |
| Discontinuity of Mind | 3,16,30 |
| Theory of Mind | 6,18,23 |
| Self | 7,19,25 |
| Planning | 1,15,22 |
| Sleepiness | 2,14,24 |
| Comfort | 4,17,26 |
| Somatic Awareness | 8,13,21 |
| Health Concern | 9,12,27 |
| Visual Thought | 10,20,28 |
| Verbal Thought | 5,11,29 |
|  |  |
| Non-factor items** | 31-49 |
| Validation items | 50-54 |

* unpublished individual items that people may want to add, e.g., disease-specific items
or new ideas for important dimensions. Note, if these items should eventually become a new factor, at least 3 items coding for that dimension is necessary

** not part of the 10-factor ARSQ 2.0 model

^1^The German translation of the Amsterdam Resting-State Questionnaire was kindly provided by Dr. Klaus Linkenkaer-Hansen, VU Amsterdam, The Netherlands. For questions about the questionnaire or translations into other languages, please contact [klaus.linkenkaer@cncr.vu.nl](mailto:klaus.linkenkaer@cncr.vu.nl)
